
JOURNAL INFORMATION
==============================
NLM Title Abbreviation: ScientificWorldJournal
Journal ID: ScientificWorldJournal
NLM Journal ID: 101131163
Journal ID: TSWJ

The Scientific World Journal

ISSN: 2356-6140
EISSN: 1537-744X
Publisher: Wiley

ARTICLE INFORMATION
==============================
PMCID: PMC5901192
PMID: 17982584
DOI: 10.1100/tsw.2007.259
Article ID: 983106
Article version: 1
Subjects: Editorial

Impulsivity and Addiction: A Tribute to Henri Begleiter

Porjesz Bernice 1 *
Li Ting-Kai 2
1 Henri Begleiter Neurodynamics Laboratory, SUNY Downstate Medical Center, Brooklyn, New York, USA
2 National Institute on Alcohol Abuse and Alcoholism, NIH, Bethesda, MD, USA
*Bernice Porjesz: Bernice.porjesz@Downstate.eduAcademic Editors: D. Shurtleff and S. Ferre

Publication date: 2007
Electronic publication date: 2007 Nov 2
Volume: 7
First page: 121
Last page: 123
Received 2007 Sep 6; Accepted 2007 Sep 6
Copyright: Copyright © 2007 Bernice Porjesz and Ting-Kai Li.
Copyright year: 2007
License: This is an open access article distributed under the Creative Commons Attribution License, which permits unrestricted use, distribution, and reproduction in any medium, provided the original work is properly cited.
License URL: https://creativecommons.org/licenses/by/3.0/

==============================
Editorial
NIDA Special Issue on Frontiers in Addiction Research
*TheScientificWorldJOURNAL* (2007) 7(S2), 121–123
ISSN 1537-744X; DOI 10.1100/tsw.2007.259

TheScientificWorldJOURNAL
www.thescientificworld.com

### Impulsivity and Addiction: A Tribute to Henri Begleiter

Bernice Porjesz[1] and Ting-Kai Li[2]
[1]*Henri Begleiter Neurodynamics Laboratory, SUNY Downstate Medical Center, Brooklyn, New York;* [2]*National Institute on Alcohol Abuse and Alcoholism, NIH, Bethesda, MD*

**E-mail:** Bernice.porjesz@Downstate.edu

*Received September 6, 2007; Accepted September 6, 2007; Published November 2, 2007*

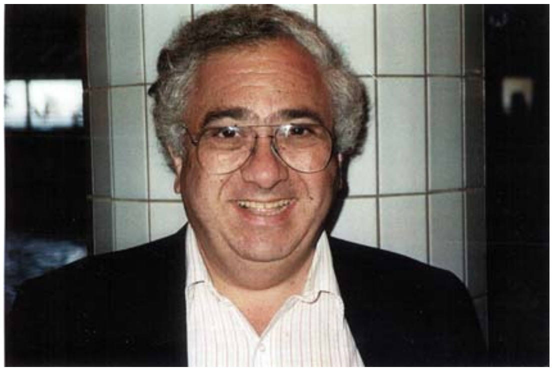

The 2007 NIDA-NIAAA Symposium, part of NIDA's Mini-Convention on Frontiers in Addiction Research, chaired by Drs. Nora Volkow and T.K. Li, and featuring speakers K. Kendler, B. Porjesz, and D. Goldman, was held in honor of Henri Begleiter, Distinguished Professor of Psychiatry and Neuroscience at SUNY Downstate Medical Center, who passed away on April 6, 2006. Henri was an exceptional scientist of international acclaim, whose innovative approaches to research and scientific vision were an inspiration to us all. He was one of the truly great, wise, and charismatic leaders in the field, who possessed incredible professional and personal gifts. He enjoyed reading voraciously on wide-ranging topics, always in pursuit of new knowledge and exciting ideas. He had the astonishing ability to integrate information from a wide variety of fields. With his scientific vision and encyclopedic knowledge in multiple fields, he almost single-handedly brought together the fields of neurophysiology, genetics, and alcoholism, and his ideas are the basis and inspiration for this symposium. Henri very much lived in accordance with his favorite quote from Louis Pasteur, which he hung in large letters over his desk:

> *"Dans les champs de l'observation, l´hazard ne favorise que les esprits préparés"*
> *[When it comes to observation, chance only favors the prepared minds.]*

Henri's research path manifested incredible vision — progressing from early animal and human work studying underlying brain hyperexcitability related to alcoholism[1,2], to subsequent findings demonstrating that this brain excitability is critically involved in the genetic predisposition toward the development of alcoholism, substance abuse, conduct disorder, and antisocial personality disorder — an amalgam of disorders now known as externalizing disorders. His pioneering approach of directly applying

*Corresponding author.
©2007 with author.
Published by TheScientificWorld; www.thescientificworld.com rat and monkey findings to human research was several decades before the now-popular translational research approach.

Starting with the ground-breaking finding, published in *Science*, that some neurophysiological anomalies in alcoholics were already present in their young offspring before any exposure to alcohol and drugs[3], Henri proposed a model that changed the thinking in the field; namely, rather than a consequence of alcoholism, this neural hyperexcitability was a predisposing factor leading to the development of alcoholism and related disorders[4]. This innovative study was replicated throughout the world and launched him on a systematic search to elucidate the genes underlying this predisposition.

With his foresight and charismatic leadership, Henri was instrumental in organizing the large Collaborative Study on the Genetics of Alcoholism (COGA), which he headed since its inception 18 years ago. From the beginning, Henri placed much emphasis on the use of endophenotypes to aid in the search for genes, which was ahead of its time when it was proposed. His approach of using brain oscillations as endophenotypes proved successful in identifying genes involved in the predisposition to develop alcoholism and related disorders, and is still state-of-the-art today. One major finding was the discovery that the *GABRA2* receptor gene is involved in human EEG beta oscillations as well as predisposition for alcoholism and related disorders[5,6,7,8]. These findings have recently been replicated in several laboratories[9,10,11,12]. Another COGA finding based on this approach was that a cholinergic muscarinic receptor gene (*CHRM2*) is involved in theta and delta oscillations underlying P300, and that the same gene is also associated with alcoholism and depression[13,14,15].

Endophenotypes are proximal to gene function and, hence, provide a powerful approach in uncovering genes and genetic risk factors contributing to complex behavioral phenotypes[16,17,18,19], such as alcohol dependence and drug abuse. Recent evidence suggests that alcohol dependence is part of a spectrum of disinhibitory disorders, which include externalizing and substance use disorders. Many of the same genetic risk factors underlie these disinhibitory co-occurring disorders and can be explained by a small number of common underlying genetic factors[20]. This symposium will address how some genetically influenced differences in susceptibility are unique to alcoholism while others, such as impulsivity, influence a range of related outcomes including externalizing and mood disorders and substance abuse.

### REFERENCES

1. Begleiter, H. and Porjesz, B. (1977) Persistence of brain hyperexcitability following chronic alcohol exposure in rats. *Adv. Exp. Med. Biol*. **85B,** 209–222.
2. Begleiter, H., Porjesz, B., and Yerre-Grubstein, C. (1974) Excitability cycle of somatosensory evoked potentials during experimental alcoholization and withdrawal. *Psychopharmacologia* **37,** 15–21.
3. Begleiter, H., Porjesz, B., Bihari, B., and Kissin, B. (1984) Event-related brain potentials in boys at risk for alcoholism. *Science* **225,** 1493–1496.
4. Begleiter, H. and Porjesz, B. (1999) What is inherited in the predisposition toward alcoholism? A proposed model. *Alcohol. Clin. Exp. Res.* **23,** 1125–1135.
5. Porjesz, B., Almasy, L., Edenberg, H.J., Wang, K., Chorlian, D.B., Foroud, T., Goate, A., Rice, J., O'Connor, S.J., Rohrbaugh, J., Kuperman, S., Bauer, L.O., Crowe, R., Schuckit, M., Hesselbrock, V., Conneally, P.M., Tischfield, J.A., Li, T.K., Reich, T., and Begleiter, H. (2002) Linkage disequilibrium between the beta frequency of the human EEG and a GABAA receptor gene locus. *Proc. Natl. Acad. Sci. U. S. A.* **99,** 3729–3733.
6. Edenberg, H.J., Dick, D.M., Xuei, X., Tian, H., Almasy, L., Bauer, L.O., Crowe, R., Goate, A., Hesselbrock, V., Jones, K.A., Kwon, J., Li, T.K., Nurnberger, J.I., Jr., O'Connor, S.J., Reich, T., Rice, J., Schuckit, M., Porjesz, B., Foroud, T., and Begleiter, H. (2004) Variations in GABRA2, encoding the a2 subunit of the GABA-A receptor are associated with alcohol dependence and with brain oscillations. *Am. J. Hum. Genet*. **74,** 705–714.
7. Agrawal, A., Edenberg, H.J., Foroud, T., Bierut, L., Dunne, G., Hinrichs, A.L., Nurnberger, J.I., Jr., Crowe, R., Kuperman, S., Schuckit, M., Begleiter, H., Porjesz, B., and Dick, D.M. (2006) Association of GABRA2 with drug dependence in the collaborative study of the genetics of alcoholism sample. *Behav. Genet*. **36(5),** 640–650.
8. Dick, D.M., Bierut, L., Hinrichs, A.L., Fox, L., Bucholz, K.K., Kramer, J.R., Kuperman, S., Hesselbrock, V., Schuckit, M., Almasy, L., Tischfield, J.A., Porjesz, B., Begleiter, H., Nurnberger, J.I., Jr., Xuei, X., Edenberg, H.J., and Foroud, T. (2006) The role of GABRA2 in risk for conduct disorder and alcohol and drug dependence across developmental stages. *Behav. Genet*. **36,** 577–590.

9. Covault, J., Gelernter, J., Hesselbrock, V., Nellissery, M., and Kranzler, H.R. (2004) Allelic and haplotypic association of GABRA2 with alcohol dependence. *Am. J. Med. Genet. B Neuropsychiatr. Genet.* **129B,** 104–109.
10. Lappalainen, J., Krupitsky, E., Remizov, M., Pchelina, S., Taraskina, A., Zvartau, E., Somberg, L.K., Covault, J., Kranzler, H.R., Krystal, J., and Gelernter, J. (2005) Association between alcoholism and gamma-amino butyric acid alpha2 receptor subtype in a Russian population. *Alcohol. Clin. Exp. Res.* **29,** 493–498.
11. Fehr, C., Sander, T., Tadic, A., Lenzen, K.P., Anghelescu, I., Klawe, C., Dahmen, N., Schmidt, L.G., and Szegedi, A. (2006) Confirmation of association of the GABRA2 gene with alcohol dependence by subtype-specific analysis. *Psychiatr. Genet.* **16,** 9–17.
12. Soyka, M. (2007) Replication of GABRA2 with alcohol dependence. *J. Psychiatry Res.* E pub ahead of print.
13. Jones, K.A., Porjesz, B., Almasy, L., Bierut, L., Goate, A., Wang, J.C., Dick, D.M., Hinrichs, A., Kwon, J., Rice, J.P., Rohrbaugh, J., Stock, H., Wu, W., Bauer, L.O., Chorlian, D.B., Crowe, R.R., Edenberg, H.J., Foroud, T., Hesselbrock, V., Kuperman, S., Nurnberger, J., Jr., O'Connor, S.J., Schuckit, M., Stimus, A.T., Tischfield, J.A., Reich, T., and Begleiter, H. (2004) Linkage and linkage disequilibrium of evoked EEG oscillations with CHRM2 receptor gene polymorphisms: implications for human brain dynamics and cognition. *Int. J. Psychophysiol.* **53,** 75–90.
14. Jones, K.A., Porjesz, B., Almasy, L., Bierut, L., Dick, D., Goate, A., Hinrichs, A., Rice, P.J., Wang, C.J., Bauer, L.O., Crowe, R., Foroud, T., Hesselbrock, V., Kuperman, S., Nurnberger, J., Jr., O'Connor, S.J., Rohrbaugh, J., Schuckit, M.A., Tischfield, J., Edenberg, H.J., and Begleiter, H. (2006) A cholinergic receptor gene (CHRM2) affects event-related oscillations. *Behav. Genet.* **36(5),** 627–639.
15. Wang, J.C., Hinrichs, A.L., Stock, H., Budde, J., Allen, R., Bertelsen, S., Kwon, J.M., Wu, W., Dick, D.M., Rice, J., Jones, K., Nurnberger, J.I., Jr., Tischfield, J., Porjesz, B., Edenberg, H.J., Hesselbrock, V., Crowe, R., Schuckit, M., Begleiter, H., Reich, T., Goate, A.M., and Bierut, L.J. (2004) Evidence of common and specific genetic effects: association of the muscarinic acetylcholine receptor M2 (*CHRM2*) gene with alcohol dependence and major depressive syndrome. *Hum. Mol. Genet.* **13,** 1903–1911.
16. Gottesman, I.I. and Gould, T.D. (2003) The endophenotype concept in psychiatry: etymology and strategic intentions. *Am. J. Psychiatry* **160,** 636–645.
17. Gottesman, I. and Shields, J. (1972) *Schizophrenia and Genetics: A Twin Study Vantage Point.* Academic Press, New York.
18. Gottesman, I.I. and Shields, J. (1973) Genetic theorizing and schizophrenia. *Br. J. Psychiatry* **122,** 15–30.
19. Tsuang, M.T. and Faraone, S.V. (2000) The frustrating search for schizophrenia genes. *Am. J. Med. Genet.* **97,** 1–3.
20. Kendler, K.S., Prescott, C.A., Myers, J., and Neale, M.C. (2003) The structure of genetic and environmental risk factors for common psychiatric and substance use disorders in men and women. *Arch. Gen. Psychiatry* **60,** 929–937.

**This article should be cited as follows:**

Porjesz, B. and Li, T.K. (2007) Impulsivity and addiction: a tribute to Henri Begleiter. *TheScientificWorldJOURNAL* **7(S2)**, 121–123. DOI 10.1100/tsw.2007.259.
